# A Network Approach to Predict Pathogenic Genes for *Fusarium graminearum*

**DOI:** 10.1371/journal.pone.0013021

**Published:** 2010-10-04

**Authors:** Xiaoping Liu, Wei-Hua Tang, Xing-Ming Zhao, Luonan Chen

**Affiliations:** 1 Institute of Systems Biology, Shanghai University, Shanghai, China; 2 School of Communication and Information Engineering, Shanghai University, Shanghai, China; 3 National Key Laboratory of Plant Molecular Genetics, Institute of Plant Physiology and Ecology, Chinese Academy of Sciences, Shanghai, China; 4 Key Laboratory of Systems Biology, SIBS-Novo Nordisk Translational Research Centre for PreDiabetes, Shanghai Institutes for Biological Sciences, Chinese Academy of Sciences, Shanghai, China; University of Glasgow, United Kingdom

## Abstract

*Fusarium graminearum* is the pathogenic agent of *Fusarium* head blight (FHB), which is a destructive disease on wheat and barley, thereby causing huge economic loss and health problems to human by contaminating foods. Identifying pathogenic genes can shed light on pathogenesis underlying the interaction between *F. graminearum* and its plant host. However, it is difficult to detect pathogenic genes for this destructive pathogen by time-consuming and expensive molecular biological experiments in lab. On the other hand, computational methods provide an alternative way to solve this problem. Since pathogenesis is a complicated procedure that involves complex regulations and interactions, the molecular interaction network of *F. graminearum* can give clues to potential pathogenic genes. Furthermore, the gene expression data of *F. graminearum* before and after its invasion into plant host can also provide useful information. In this paper, a novel systems biology approach is presented to predict pathogenic genes of *F. graminearum* based on molecular interaction network and gene expression data. With a small number of known pathogenic genes as seed genes, a subnetwork that consists of potential pathogenic genes is identified from the protein-protein interaction network (PPIN) of *F. graminearum*, where the genes in the subnetwork are further required to be differentially expressed before and after the invasion of the pathogenic fungus. Therefore, the candidate genes in the subnetwork are expected to be involved in the same biological processes as seed genes, which imply that they are potential pathogenic genes. The prediction results show that most of the pathogenic genes of *F. graminearum* are enriched in two important signal transduction pathways, including G protein coupled receptor pathway and MAPK signaling pathway, which are known related to pathogenesis in other fungi. In addition, several pathogenic genes predicted by our method are verified in other pathogenic fungi, which demonstrate the effectiveness of the proposed method. The results presented in this paper not only can provide guidelines for future experimental verification, but also shed light on the pathogenesis of the destructive fungus *F. graminearum*.

## Introduction

The filamentous ascomycete *Fusarium graminearum* (teleomorph *Gibberella zeae*) is the major pathogenic agent of *Fusarium* head blight(FHB) [1], which can cause diseases for wheat, barley and other crops, and is becoming a serious disease in many countries over the world. In general, FHB causes diseases to crops within a few weeks [2], and results in huge economic loss and causes health problems to human and animals by contaminating grains [3]. For example, in the United State and Europe, *F. graminearum* reduces crop yield significantly and contaminates the grains with trichothecene mycotoxins, such as deoxynivalenol and nivalenol toxin [4]. Therefore, it is necessary to understand the pathogenesis of *F. graminearum* by dissecting the components involved in the pathogenic procedure, *i.e.* pathogenic genes, thereby preventing the invasion of this destructive fungus into crops. In this paper, the definition of pathogenic genes is adopted from plant pathology, where pathogenic genes are those that result in a loss or reduction in disease symptoms when disrupted [5]. The pathogenic genes can be identified in lab by techniques, such as gene knockout or silencing. By the writing of this paper, there are 49 pathogenic genes of *F. graminearum* that were verified by biological experiments and stored in PHI-base database (http://www.phi-base.org/query.php). However, the pathogenic gene list is far from complete and it will be a painful process to identify pathogenic genes in lab considering the genome size of *F. graminearum* and time-consuming experiments. On the other hand, computational methods can provide alternative ways for this problem, especially after the genome sequence of *F. graminearum* is released by Broad Institute (http://www.broadinstitute.org). In literature, comparative genomics method tries to predict pathogenic genes by comparing pathogenic and non-pathogenic fungi [6]. However, it is found that there are no specific genes that uniquely occur in pathogenic fungi but not in non-pathogenic fungi, which makes it difficult to identify pathogenic genes of *F. graminearum*.

Based on the observations of pathogenicity of model pathogens [7], it is believed that the pathogenesis of *F. graminearum* involves a complex network of proteins and other molecules, including those that might be secreted into host cells. Therefore, the molecular interaction network of *F. graminearum* can provide insights into the pathogenesis of the destructive fungus. Recently, the protein-protein interaction map was delineated for *F. graminearum* in our previous work [8], which can give hints to potential pathogenic genes that work in concert in the pathogenesis procedure. Furthermore, the pathogenic genes are generally differentially expressed before and after the pathogen invading its host so that the pathogen can successfully break through its host immune system and adopt its life inside the host. That is, the genes of *F. graminearum* that are differentially expressed before and after the invasion of this destructive pathogen may be pathogenic genes. However, differentially expressed genes alone may lead to false positives while identifying key genes involved in disease procedure because some genes are not involved in the pathway of pathogenic genes even though they show significant expression changes. In addition, in the literature, it was found that the integration of protein interaction and gene expression is useful to identify the biological processes induced by specific perturbations, *e.g.* drug [9] or extracellular stimuli [10].

In this paper, a novel systems biology approach is presented to predict pathogenic genes for *F. graminearum* by integrating protein interaction map and gene expression data. With the assumption that interacting proteins usually share similar functions due to “Association rule” [11] and are possibly involved in the same pathway [10], a pathogenic subnetwork that consists of potential pathogenic genes is identified with a small number of known pathogenic genes as seed genes. The genes in the subnetwork are further required to be differentially expressed before and after the invasion of the pathogenic fungus. Therefore, the candidate genes in the subnetwork are expected to be involved in the same biological processes as seed genes, and thereby may be pathogenic genes. The prediction results show that most of pathogenic genes of *F. graminearum* are enriched in two important signal transduction pathways, including G protein coupled receptor pathway and MAPK signaling pathway, which are known related to pathogenesis in other fungi [5]. In addition, the orthologs of several pathogenic genes predicted by our method are verified in other pathogenic fungi, which demonstrate the effectiveness of the proposed method. It is believed that our predictions can provide guidelines for future experimental verification, and shed light on the pathogenesis of the destructive fungus *F. graminearum*.

## Results

### Detection of differentially expressed genes

In general, some genes are differentially expressed in the infection procedure of pathogenic fungus. For example, some enzymes are over-expressed and highly produced to destroy the host's defense system so that the *F. graminearum* can invade the host successfully. Therefore, the differentially expressed genes are possibly related to the interaction between the pathogen and its host, and thereby may be pathogenic genes. At present, there are no gene expression data of *F. graminearum* that are measured before and after its invasion in the same experiments. In this work, the microarray data obtained with *F. graminearum* Affymetrix GeneChip were downloaded from Plant Expression Database (PLEXdb, http://www.plexdb.org/index.php), which is a unified public resource for gene expression data of plants and plant pathogens. Table 1 lists the gene expression data sets and corresponding conditions under which the data were collected, including the expression data before and after the invasion of *F. graminearum*.

**Table 1:** Gene expression data of *F. graminearum*.

	Accession No	Condition	Biological replicates
After invasion	FG1	24 hours after inoculated	3
	FG1	48 hour safter inoculated	3
	FG1	72 hours after inoculated	3
	FG1	96 hours after inoculated	3
	FG1	144 hours after inoculated	3
	FG12	2 dpi after inoculated	4
	FG12	14 dpi after inoculated	4
	FG12	35 dpi after inoculated	3
Before invasion	FG2	Complete Media	3
	FG2	Carbon Starvation	3
	FG2	Nitrogen Starvation	3
	FG4	Complete Media	1
	FG7	2 hours after conidia germination	3
	FG7	8 hours after conidia germination	3
	FG7	24 hours after conidia germination	3
	FG12	mycelia culture condition	4
	FG10	Complete Media	3

The gene expression data were divided into two groups, *i.e.* before invasion and after invasion, based on the experimental conditions under which the expression data were generated. The detailed descriptions of experimental conditions can be found in PLEXdb (http://www.plexdb.org/).

After the gene expression data were obtained, the Mann-Whitney Wilcoxon test was utilized to identify those genes that were differentially expressed before and after the invasion of *F. graminearum*. The Wilcoxon test is a non-parametric rank-based test and was used here because there is no prior information about the distribution underlying the microarray data. As a consequence, there are 7,267 genes in total that were chosen from 13,367 genes of *F. graminearum* with a 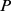-value threshold of 0.01.

### Identification of pathogenic network

The differentially expressed genes can give hints on pathogenesis of the destructive pathogen. However, the differentially expressed genes alone may lead to false positives because some genes show significant expression difference due to some stimuli but are not related to the pathogenic procedure. In general, the pathogenesis of pathogenic fungus involves a complex network of proteins and other molecules, including those that might be secreted into host cells. In other words, a number of genes will be regulated to respond to the stimuli in the pathogenesis procedure, where these genes work in concert so that *F. graminearum* can successfully break through the immune system of the host. Therefore, the protein-protein interaction (PPI) information can provide insights into the pathogenesis of *F. graminearum*. Recently, the interactome map of *F. graminearum* [8] was delineated in our previous work and the PPI database, namely FPPI, is freely accessible (http://csb.shu.edu.cn/fppi).The details about predicting protein interactions for *F. graminearum* can be found in [8]. In this work, the core PPI data set that consists of 27,102 high-confidence interactions among 3,745 proteins was used. In literature, a small number of genes have been identified as pathogenic genes, *e.g.* 49 *F. graminearum* genes from PHI-base (Version 3.1) were verified to be pathogenic genes. With the assumption that interacting proteins generally share similar functions and are involved in similar biological processes [11], the genes that interact with known pathogenic genes are possibly pathogenic genes. With known pathogenic genes as seed genes, a subnetwork was extracted from the *F. graminearum* protein interaction network, where the genes in the subnetwork interact with at least one seed gene. In this work, the 49 pathogenic genes from PHI-base were used as seed genes, among which 20 genes can be mapped to *F. graminearum* interaction map. As a result, 479 interactions were identified to link to at least one seed gene, and these interactions involve 294 genes in total.

**Figure 1:**
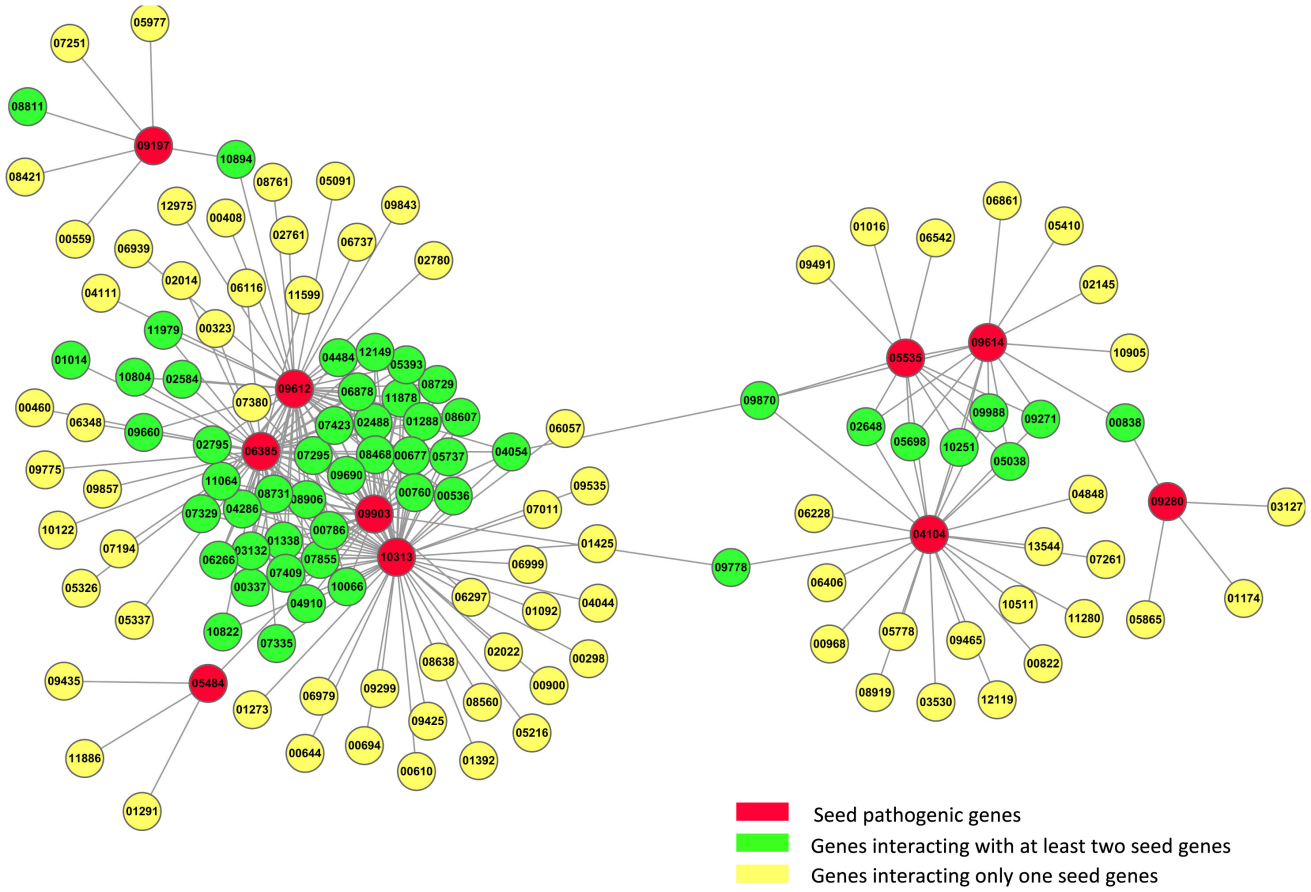
The pathogenic network. The red vertices denote seed genes from PHI-base, *i.e.* the known pathogenic genes, the green vertices denote genes that interact with at least two seed genes, and the yellow vertices denote genes that interact with only one seed gene. For clarity, the prefix “FGSG_” was omitted from gene names. The subnetwork consists of 127 genes and 259 interactions. Furthermore, the genes in the subnetwork are differentially expressed before and after the invasion of *F. graminearum*. Note that vertices 01014 and 08811 actually connect two seed genes, and those seed genes with less significant expression changes were discarded.

Although the genes interacting with seed genes are possibly pathogenic genes, they may also just interact with seed genes to maintain the essential biological processes for *F. graminearum*. Therefore, the integration of differentially expressed genes and the subnetwork identified above can help to reduce false positives because it is believed that the expression changes of differentially expressed genes are possibly caused by the interactions with seed genes. By mapping differentially expressed genes to the subnetwork described above, we finally obtained a subnetwork that consists of 127 genes except seed genes and 259 interactions. The subnetwork that consists of seed genes and those interacting with the seed genes is shown in Figure 1, where the genes are differentially expressed before and after the invasion of *F. graminearum*, and the the prefix “FGSG_” was omitted from gene names for clarity.

**Figure 2:**
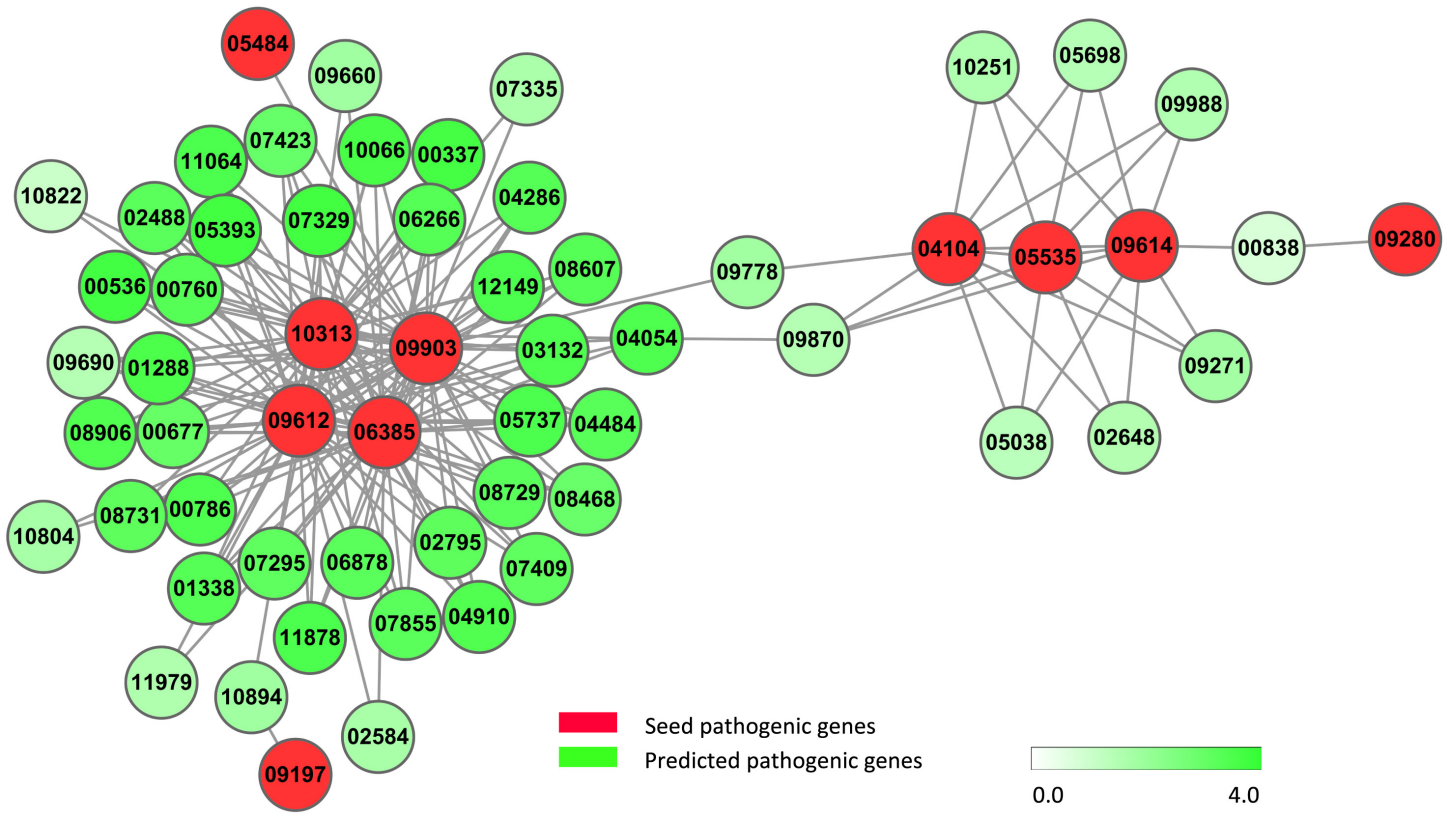
The filtered pathogenic network. The red vertices denote seed genes, *i.e.* the known pathogenic genes, the green vertices are genes that interact with at least two seed genes, and each vertex is assigned a weight. The color bar represents the relationship between color and weight, where the deeper the color is the larger the weight is. For clarity, the prefix “FGSG_” was omitted from gene names.

Furthermore, the genes that interact with at least two seed genes were identified because these genes are more likely to be pathogenic genes due to their tight interactions with the seed genes. Figure 2 shows the subnetwork that consists of only genes with at least two interactions with seed genes, and this subnetwork is called pathogenic network hereafter. Interestingly, it is found that four seed genes, *i.e.* FGSG_09612, FGSG_09903, FGSG_06385 and FGSG_10313, interact with each other and form a clique. Therefore, these four seed genes may belong to the same complex or pathway that is involved in the pathogenic procedure. Accordingly, the genes that interact with these four seed pathogenic genes are more likely to be pathogenic genes. For example, FGSG_06878 is a calcium/calmodulin-dependent protein kinase, and this enzyme regulates ion homeostasis and cell wall construction and affects fungus virulence in many fungi [12]. FGSG_00786 belongs to Serine/Threonine-protein kinase family, and regulates many intracellular metabolic processes including the control of cell growth and division [13].

In addition, two tightly interconnected modules can be found in the pathogenic network with maxClique, a tool of RBGL package [14] of Bioconductor (http://www.bioconductor.org/), as shown in Figure 2, where one module means a subnetwork in which the vertexes are more closely and intensely linked to one another rather than to those outside of the subnetwork. The genes in each network module are possibly involved in the same regulatory or signaling pathway as seed genes, and are therefore more likely to be related to pathogenic procedure. shows the two network modules that involve at least two seed pathogenic genes, where the annotations for these genes were downloaded from MIPS Fusarium graminearum Genome DataBase (FGDB, http://mips.helmholtz-muenchen.de/genre/proj/fusarium/). In addition, the annotations of genes in the pathogenic network were investigated by looking at the descriptions of these genes from MIPS FGDB, and it was found that most of the genes are involved in two signaling pathways, including G-protein coupled receptor signaling pathway and mitogen-activated protein kinase (MAPK) cascades signaling pathways. In literature, it has been found that these two pathways are related to pathogenesis [5].

**Figure 3:**
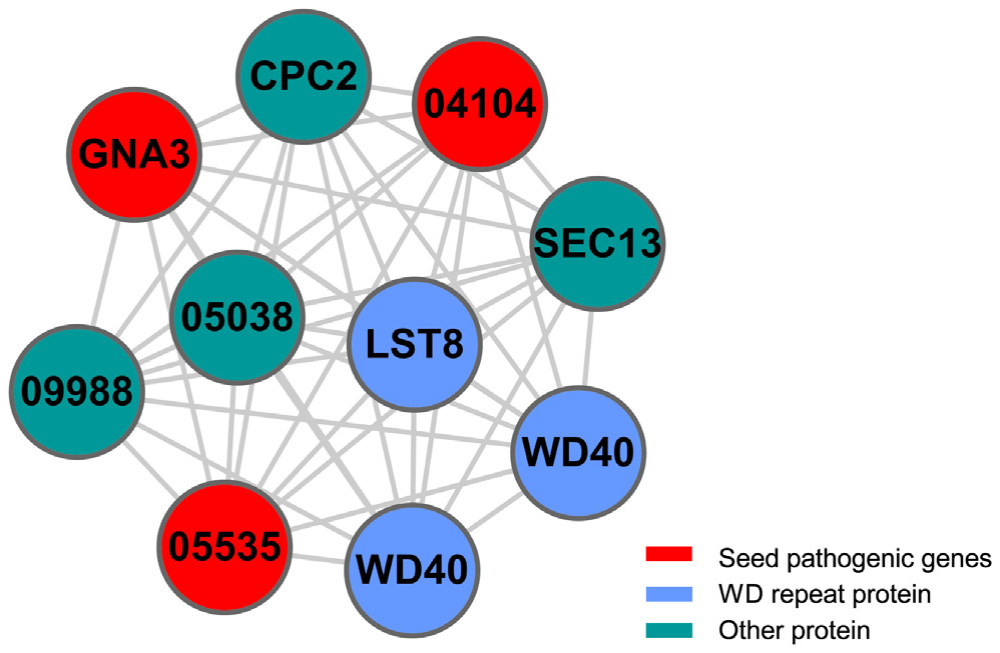
Module one. The red vertices denote seed genes, *i.e.* the known pathogenic genes, and other vertices denote genes that interact with seed genes. Some genes are not annotated in MIPS, and the original name was used, such as FGSG_05038. All the genes in the module were colored according to their functions. For clarity, the prefix “FGSG_” was omitted from gene names in the figure.

In module one, there are 10 genes that form a fully connected subnetwork and 3 seed genes are involved as shown in Figure 3. From Table S1, we can see that most of the genes in module one belong to the G protein-linked signal transduction pathway, including G protein family members FGSG_04104, FGSG_05535, FGSG_09614, and FGSG_09988. Especially, FGSG_04104 is the 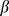 subunit of guanine nucleotide-binding, and interacts with three G protein 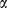 subunits, FGSG_05535, FGSG_09614 and FGSG_09988. These 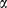 subunits of G protein are able to activate three or more effectors which in turn transmit the signals to several transcription factors and initiate more than one transcription process. Furthermore, there are some important regulator proteins in module one, such as LST8(FGSG_10251) and CPC2(FGSG_09870), and protein transport proteins, such as SEC13(FGSG_09271). SEC13 protein is related to vesicle biogenesis from endoplasmic reticulum during the transportation of proteins [15]–[17], where vesicular trafficking is the main way for protein secretion and is also the main track for exoenzyme secretion by secretory vesicle. That is, SEC13 is probably involved in the process of transmembrane transport of extracellular hydrolytic enzyme. LST8(FGSG_10251) protein is a WD-repeat protein and also a negative regulator of some transcription factors [18], and acts as a scaffold in the signaling pathway to receive signals from upstream and regulate downstream gene expression. LST8 is also a component of TOR (the target of rapamycin) [19], which is a phosphatidylinositol kinase-related protein kinase (PIKK) that controls cell growth in response to nutrients [19] and plays important roles in virulence-associated traits of several fungal pathogens [20]. CPC2(FGSG_09870) is an adaptor to favor protein kinase C (PKC)-mediated phosphorylation and subsequent activation of c-Jun NH2-terminal kinase [21]. CPC2 positively regulates the synthesis of the stress-responsive transcription factor ATF1 [21], whereas ATF1 and c-Jun are two important transcription factors that enable a number of crucial metabolism processes [22]. It was found that *cpc2* is a pathogenic gene involved in invasive growth in response to glucose limitation in *Saccharomyces cerevisiae* [23], and is also involved in the control of G2/M transition and belongs to mitogen-activated protein kinase (MAPK) pathway [21]. FGSG_04054 is related to VHS1, which is a cytoplasmic Serine/Threonine protein kinase that is involved in phospho-dephosphorylation processes in *S. cerevisiae* [24]. There are some proteins in G-protein coupled receptor signaling pathway that contain WD repeat structure, such as FGSG_09271, FGSG_05698, and FGSG_02648. The WD repeat proteins are involved in diverse cellular pathways, such as signal transduction, pre-mRNA splicing, transcriptional regulation, cytoskeletal assembly, and vesicular traffic [25], [26]. In summary, module one is related to signal transduction, transcription and protein transport, and these processes are possibly related to the invasion procedure. In addition, to investigate the predicted pathogenic genes in module one, the orthologs of our predicted pathogenic genes were identified in other pathogenic fungi by utilizing Inparanoid [27]. It was found that FGSG_09988 has one orthologous gene *magC* in *Magnaporthe grisea*, and one orthologous gene *gpa2* in *Ustilago maydis*, respectively. Both *magC* and *gpa2* have been verified to be related to pathogenic processes by biological experiments [28], [29]. According to the annotations from PHI-base, it is found that both MAGC and GPA2 belong to G-protein coupled receptor protein signaling pathway, and are related to signal transducer activity. Specifically, GPA2 is involved in ascospore formation and transmitting the pheromone signal that is required for pathogenicity development [29]. Therefore, FGSG_09988 is believed to be a real pathogenic gene of *F. graminearum*.

**Figure 4:**
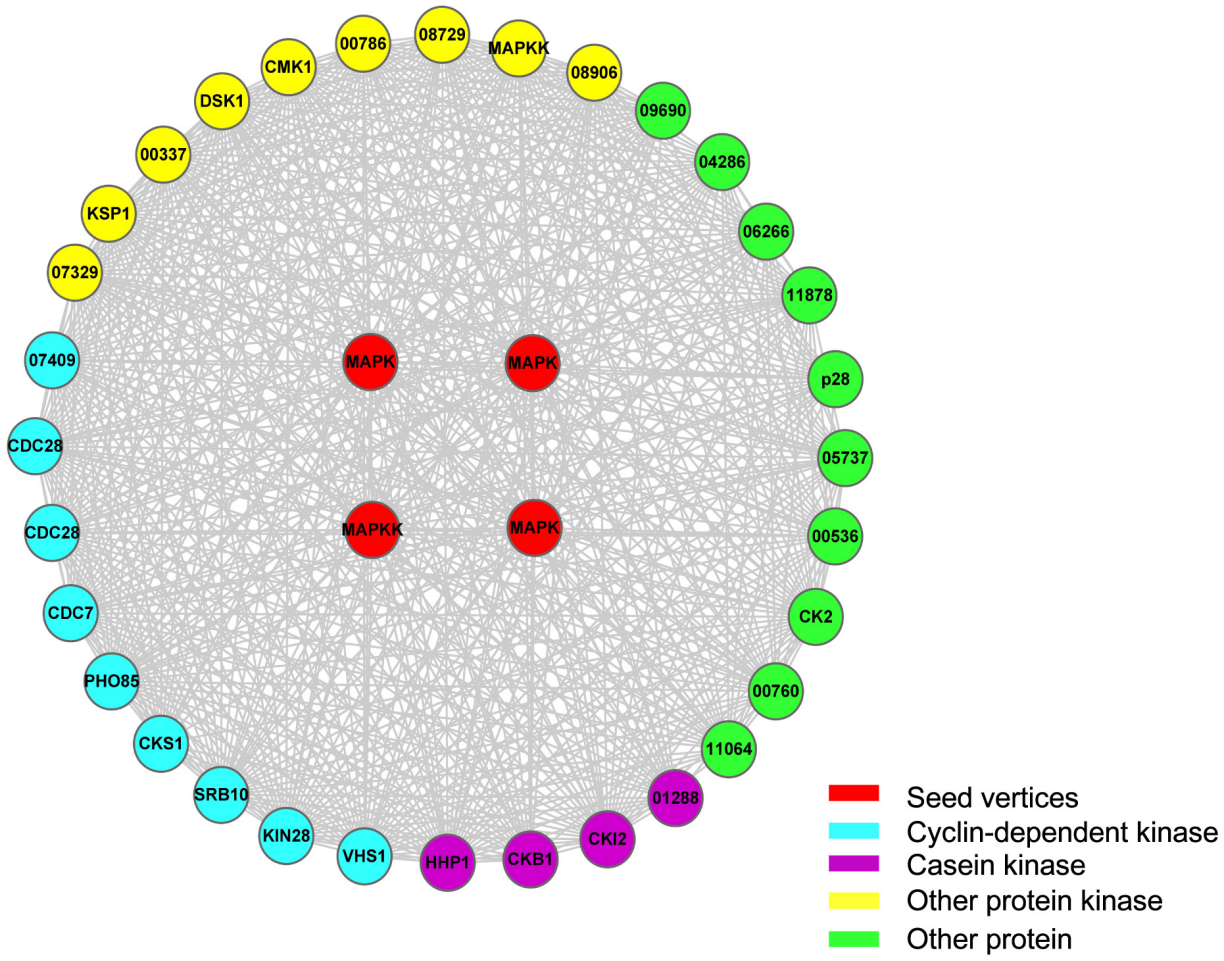
Module two. The red vertices denote seed genes, *i.e.* known pathogenic genes, and other vertices denote genes that interact with seed genes. Some genes are not annotated in MIPS, and the original name was used, such as FGSG_00337. All the genes in the module were colored according to their functions. For clarity, the prefix “FGSG_” is omitted from gene names in the figure.

Figure 4 shows module two, where there are 36 genes that form an intensely connected subnetwork with 4 seed genes involved. From Table S1, it can be seen that most genes from module two are involved in MAPK signal transduction pathway, where the pathway includes MAP kinase kinase, MAP kinase, transcription factors, kinases, and regulator proteins for RNA splicing and specific protein expression. It is interesting to see that probable PHO85, cyclin-dependent kinase (FGSG_05393) is involved in regulating the cellular responses to nutrimental and environmental conditions, and the progression through cell cycle [30], thereby possibly participates in the interaction between *F. graminearum* and its host. After the fungus breaks through the plant cell wall, it should plunder nutrient from its host for living, and accelerates the cell cycle and starts the cellular responses to nutrimental levels. FGSG_11878 is a cutinase negative acting protein, where cutinase plays an important role in pathogenesis. In general, plant organs are protected by a cuticle composed of an insoluble polymeric structural compound, *i.e.* cutin, which is a polyester composed of hydroxy and hydroxyepoxy fatty acids [31]. Plant pathogens produce extracellular degradative enzymes [32] that play important roles in pathogenesis. Cutinase is one of such enzymes, which hydrolyses cutin and enables fungus penetrating through the cuticle. Cutin monomers released from the cuticle by a small amount of cutinase on fungal spore surfaces can in turn increase the amount of cutinase secreted by the spore [31], [32]. That is, the cutinase regulated by FGSG_11878 is necessary for the invasion of pathogen. Both FGSG_08468 and FGSG_03132 are probable CDC28 cyclin-dependent protein kinase and control the persistent hyphal growth [33], while the polarized growth of mycelia is necessary for fungi to invade its host [34]. FGSG_08729 is related to cAMP-dependent protein kinase catalytic subunit family from which certain genes were found to be involved in pathogenesis in *Aspergillus fumigates* [35] and related to pathogenicity of *U. maydis* and *M. grisea* [36]. FGSG_06878 is probable CMK1 which is a 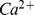/calmodulin-dependent Ser/Thr protein kinase, and was verified to be a pathogenic gene in *Colletotrichum lagenarium* and belongs to MAPK pathway [5], where the disruption of CMK1 results in *C. lagenarium* strains with impaired appressorium formation and reduced pathogenicity when inoculated into a wound [5]. FGSG_07423 is probable KIN28, which is a member of KIN protein family and is the primary kinase that phosphorylates the RNA polymerase II (RNA pol II) C-terminal domain (CTD) within a transcription initiation complex [37]. In PHI-base, some genes from KIN protein family, such as KIN1 and KIN2, are identified to be pathogenic genes in *U. maydis*. Therefore, it is possible that FGSG_07423 is involved in pathogenesis. FGSG_04484 is related to SRB10, which is a complex that is evolutionarily conserved, and integrates signals from sequence-specific activators and repressors so that the SRB10 module may sterically block the mediator interactions with RNA polymerase II to inhibit transcription [38]. FGSG_02488 is related to Dis1-suppressing protein kinase DSK1, which is an important regulator of SR proteins (Serine/Arginine-rich proteins), and SR proteins are a class of evolutionarily conserved factors important for alternative splicing, especially in pre-mRNA splicing and post-transcription regulation [39]. In addition, the orthologs of genes in module two were identified in other pathogenic fungi. It was found that the orthologous protein BcPIC5 of FGSG_09690 has already been verified in pathogen *Botrytis cinerea*, where BcPIC5(PHI:548) belongs to protein folding process with molecular function of peptidyl-prolyl *cis-trans* isomerase activity, and BcPIC5 is found related to calcineurin regulation in pathogenesis [40]. Therefore, it is believed that FGSG_09690 is a potential pathogenic gene of *F. graminearum*.

From Figure 2, we can see that there are links between module one and module two, which indicates the flow of information from G protein-linked receptor to MAPK cascade reactions. Especially, FGSG_09778 connecting both module one and module two, is probable transforming protein RAS-1 by annotation from MIPS FGDB. In literature, there are extensive evidences about biologically significant cross-talks between G protein-coupled receptors and MAPK-mediated pathways [41]. In Figure 2, FGSG_09870 interacts with FGSG_04054 which is a vertex in module two and interacts with MAPKK protein (FGSG_09903). It is known that MAPKK can be activated by G protein [22]. Therefore, the extracellular signals could be transmitted to MAPK pathway through FGSG_09870 and FGSG_04054 from G protein. The signal transduction from G protein-coupled receptors to MAP kinase involves 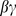 subunits of heterotrimeric G proteins acting on a RAS-dependent pathway [23], [42]. The gene from module one that connects FGSG_09778 is FGSG_04104, which is a 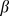 subunit of G protein, which is consistent with the results in literature [41]. From the results listed above, we speculate that the pathogenic signal is transmitted from G protein-coupled receptor pathway to MAPK signaling pathway.

### Properties of the pathogenic network

Since pathogenic genes are important for a pathogen to invade its host and assimilate nutrition from the host, the pathogenic genes should have some specific properties due to the physiological processes in which they are involved. In this work, several indices that are widely used in complex network [43]–[45] were utilized to investigate the properties of pathogenic genes, including degree distribution, clustering coefficient and betweenness. The details about how to calculate the indices can be found in MATERIALS AND METHODS. With the *F. graminearum* PPI network as the background network, the three indices were respectively obtained for the pathogenic genes and all genes in PPIN. Table 2 respectively lists the statistics for seed pathogenic genes, our predicted genes and all genes in PPIN, where the statistical number represents the average over corresponding genes. From Table 2, it can be seen that the degree and betweenness distributions of our predicted pathogenic genes are more similar to those of the seed pathogenic genes. From the degree and betweenness distributions, we can see that pathogenic genes generally connect more genes, thereby playing important roles in the biological processes. The distribution of clustering coefficients indicates that the pathogenic genes tend to be clustered together and act in concert. In other words, the pathogenic genes are more possibly involved in same pathways in which genes work together, and the pathogenesis is possibly regulated by these pathways without affecting the normal processes considering the modularity and robustness of the biological system.

**Table 2:** Properties of pathogenic network.

Genes	Degree	Clustering coefficient	Betweenness
All genes in PPI	14.2271	0.4659979	4531.312
Seed genes	23.2	0.552301	28771.23
Predicted pathogenic genes	25.83465	0.7549737	20277.26

The distribution of degree, clustering coefficient and betweenness were respectively investigated for pathogenic genes, seed genes, and all genes in PPI.

**Figure 5:**
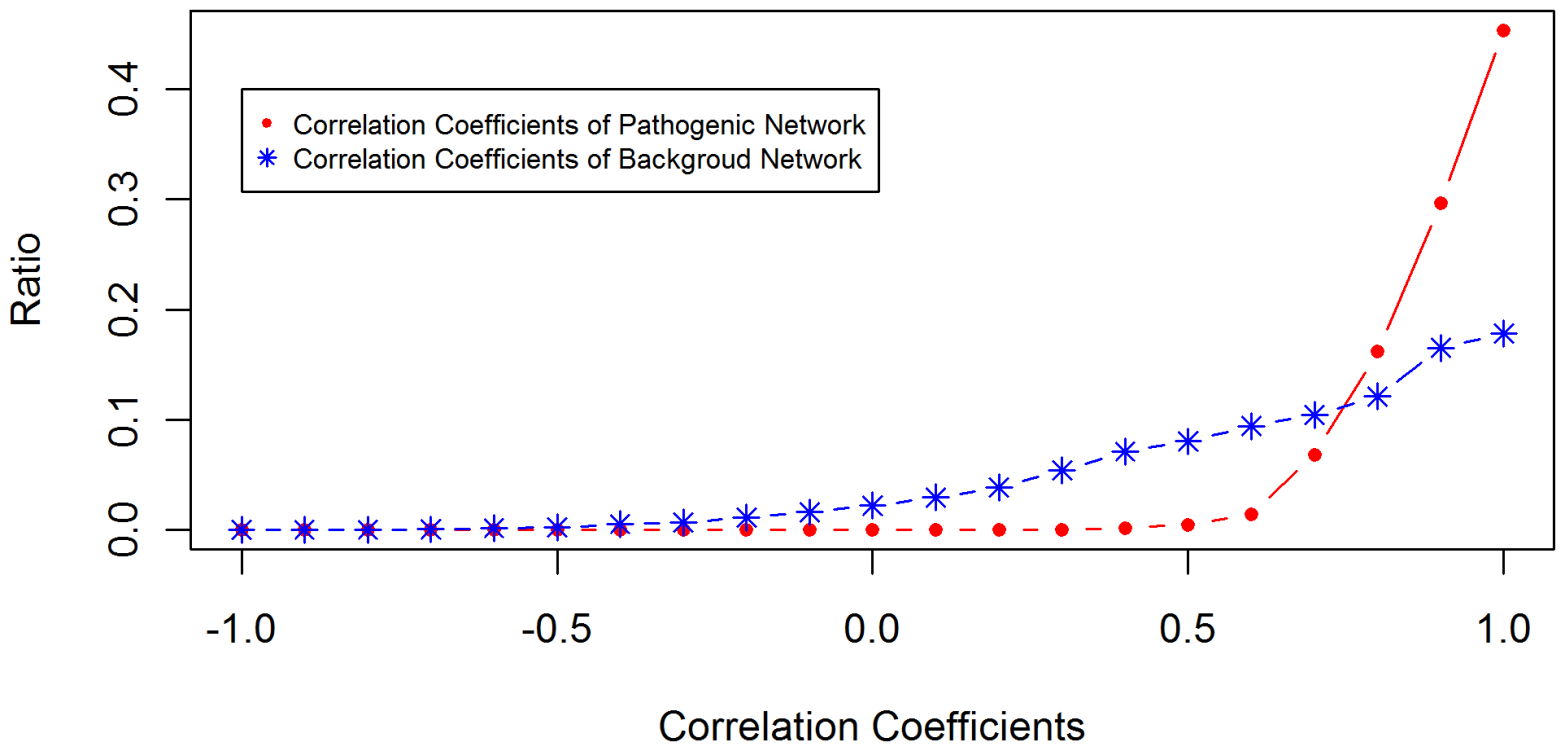
Comparison of correlation coefficients of pathogenic and background network. The distributions of correlation coefficients of pathogenic network and background network. The pathogenic network includes all our predicted pathogenic genes. The background network represents the whole core PPIN.

In addition, with the assumption that pathogenic genes work in concert for the fungus to invade the host, the pathogenic genes should co-express significantly in the invasion procedure. The *Pearson* correlation coefficients were obtained using all gene expression data, including those measured before and after the pathogen invading its host. We compared the distribution of correlation coefficients between pathogenic network and background network, as shown in Figure 5. From Figure 5, we can see that the correlation coefficients of genes in pathogenic network are obviously higher than those of the background network. In other words, the genes in pathogenic network co-express more consistently. To facilitate the biologists to choose those pathogenic genes with high confidence, each pathogenic gene was assigned a weight based on its correlations and interactions with seed genes. The details about the assignment of weight for each gene can be found in MATERIALS AND METHODS. All the genes were ranked according to the weights, where the larger the weight is, the more confident the corresponding gene is pathogenic gene. The genes were ranked in this way because one gene is more possibly a pathogenic gene if the gene interacts with more seed genes and co-expresses with seed genes. With the weights assigned, the pathogenic genes were ranked in a descending order as shown in Table S2. From Table S2, we can see that all pathogenic genes in the two modules identified above have high weights and are ranked top, thereby are more likely to be pathogenic genes because they have more interactions and higher correlations with known pathogenic genes.

### Significance analysis of pathogenic modules

To see the significance of the modules identified above, a significance score (SS) was defined for each module, where SS is defined as the geometric mean of 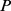-values accompanying nodes in one module and the 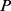-value of each node is obtained by the Mann-Whitney Wilcoxon test based on gene expression data. The differential expression derived 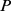-value is used here because a set of genes are more possibly involved in pathogenesis if they are tightly connected in a network and more differentially expressed. Note that a highly interconnected subnetwork does not mean that the genes in the subnetwork are significantly differentially expressed. Therefore, the SS score can be used to investigate whether a module can be detected by chance. In this work, the SS of module one is 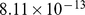 and that of module two is 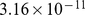.

To see the statistical significance of the two predicted modules, a 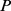-value was respectively obtained for each module by using an empirical randomization test procedure that preserves the interactions among genes, where the 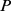-value is defined to be the probability that a module can be found with smaller SS than that of our module. The details can be found in Materials and Methods. The 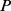-values of the two modules that we identified are respectively 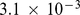 and 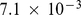, which demonstrates that the two modules are statistically significant and cannot be identified by chance.

In literature, there are a number of tools that were developed to detect modules in a complex network. To see the significance of the identified modules, a popular tool, namely MCODE [46], that is developed to detect modules in a protein interaction network was utilized to identify modules in the pathogenic network. As a result, only one module (See Figure S1) that contains at least two known pathogenic genes was found. The module that MCODE found contains the predicted module two but without any genes from the predicted module one. The SS of the module obtained by MCODE is 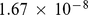, which is far larger than 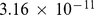 of the module two. The simple comparison of modules detected by us and MCODE demonstrates that the integration of gene expression and protein interaction indeed helps to detect modules related to pathogenesis, where MCODE only exploits protein interaction information. Note that our aim is not to develop a new module identification method. Actually, any popular methods for identifying modules can be used here as long as it can utilize the information of both gene expression and protein interaction.

### Statistical analysis of predicted pathogenic genes

In our predictions, there are 39 genes that were predicted to be potential pathogenic genes. To validate our predictions, we checked the orthologous genes of these 39 genes in other phytopathogenic fungi. As a result, the orthologous genes of FGSG_09988 in *M. grisea* (magC) and *U. maydis* (gpa2), and the orthologous gene of FGSG_09690 in *B.cinerea* (BcPIC5) were identified to be pathogenic genes [28], [29], [40]. Therefore, these two genes are believed to be pathogenic genes in *F. graminearum*.

In addition, the two verified genes FGSG_09988 and FGSG_09690 were used to see the statistical significance of our predictions. Since we have 39 predictions, for FGSG_09988, we randomly chose 39 genes respectively from *F. graminearum*, *M. grisea*, and *U. maydis*, and calculated the probability that one randomly chosen gene has at least one orthologous gene that is also a pathogenic gene in both *M. grisea* and *U. maydis*. This procedure was repeated 100000 times and the 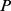-value is less than 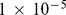. Similarly, for FGSG_09690, we randomly chose 39 genes separately from *F. graminearum* and *B. cinerea*, and calculated the probability that one randomly chosen gene has at least one orthologous gene that is also pathogenic gene in *B. cinerea*. This procedure was repeated 100000 times and the 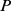-value is 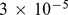.

Although there are possible false positives in our predictions, the statistical analysis of the two verified genes proves the predictive power of the proposed network biology method. We believe that our predictions can provide guidelines for future biological experiments.

## Discussion

*Fusarium graminearum* is the pathogenic agent of *Fusarium* head blight (FHB) which is a destructive disease on wheat and barley. Identifying pathogenic genes of *F. graminearum* can help to avoid economic loss and help to improve food quality. In this work, we presented a novel network approach to predict pathogenic genes with prior information of known pathogenic genes, where the genes that interact with the known pathogenic genes are candidate pathogenic genes with the assumption that interacting proteins generally share similar functions. Furthermore, the differentially expressed genes of *F. graminearum* before and after its infection were identified. A pathogenic subnetwork was then extracted by integrating differentially expressed genes and protein-protein interaction network, where the genes in the subnetwork are differentially expressed and interact with known pathogenic genes.

In addition, two intensely interconnected network modules were extracted from the network, where each module contains at least one known pathogenic gene. Further investigations into the two network modules disclosed that the network modules are respectively enriched in two signaling pathways, where module one is enriched in G-protein coupled receptor pathway and module two is enriched in MAPK signaling pathway. It is possible that the signal is transmitted from G protein coupled receptor to these two different pathways after *F. graminearum* touches its host and interacts with plant surface ligand to start the cellular signal transduction. For the G-protein coupled receptor pathway, the signal is transmitted by G protein 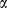 subunit through middle regulator proteins, *e.g.* protein kinases, to transcription factors which enable downstream gene transcription, or initiates particular cellular responses. For MAPK signaling pathway, the signal is transmitted by G protein 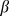 subunit to RAS protein which in turn activates the MAP kinase and downstream MAPK signaling pathway.

It is believed that module two is more important than module one in pathogenesis since module two includes more pathogenic genes and is involved in important pathogenic processes, such as nutrimental response, environmental response and cell wall degradation process. From Figure 2, we can see that the signal is possibly transmitted from module one to module two through FGSG_09778 (RAS), which connects FGSG_09903(MAPKK in module two) and FGSG_04104(G protein 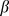 subunit in module one). It is observed that the MAPK signal transduction pathway is usually activated by RAS and heterotrimeric G proteins [22], and RAS could be regulated by heterotrimeric G protein [42]. Therefore, these two network modules are possibly involved in pathogenesis and the genes in these two modules are potential pathogenic genes. Although there are many components involved in a signaling pathway, it is possible that only the genes in our predicted modules are closely related to pathogenesis while others are not affected due to the modularity and robustness of biological systems [5]. In addition, several pathogenic genes predicted by our method were verified in other pathogenic fungi, which demonstrate the effectiveness of the proposed method.

In this work, an existing tool maxClique was employed to detect modules from PPIN. Note that our aim is not to develop new tools for identifying subnetworks. Actually, any popular methods that detect subnetworks can be used here. The statistical analysis of both our identified modules and predicted pathogenic genes demonstrate that our prediction results are statistically significant and our predictions cannot be found by chance.

In summary, the network approach presented in this work is really effective for predicting pathogenic genes of *F. graminearum* based on protein interaction network and gene expression data. We believe that our prediction results can also provide helpful guidelines for future experiments in lab.

## Materials and Methods

### Gene expression data

The microarray data obtained with *Fusarium graminearum* Affymetrix GeneChip were downloaded from Plant Expression Database (PLEXdb, http://www.plexdb.org/index.php), which is a unified public resource for gene expression data of plants and plant pathogens. In particular, the gene expression data of *F. graminearum* measured under two distinct groups of conditions corresponding to before and after the infection of the fungus into plant were used here. The details of gene expression data were summarized in Table 1.

### Detection of differentially expressed genes

The Mann-Whitney Wilcoxon test is a non-parametric rank-based test for identifying the difference between populations with respect to their medians or means. The Mann-Whitney Wilcoxon test method does not require the sample data to be or nearly normal distribution, and therefore is less sensitive than parametric hypothesis test, such as Student's 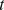-test and 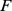-test. The Wilcoxon test is used here because it is not guaranteed that the microarray data obey normal distribution. The genes that are differentially expressed between two conditions were identified by using Wilcox.test function of R, where the genes with 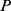-value less than 0.01 were selected for further investigation.

### Identification of pathogenic network

**Figure 6:**
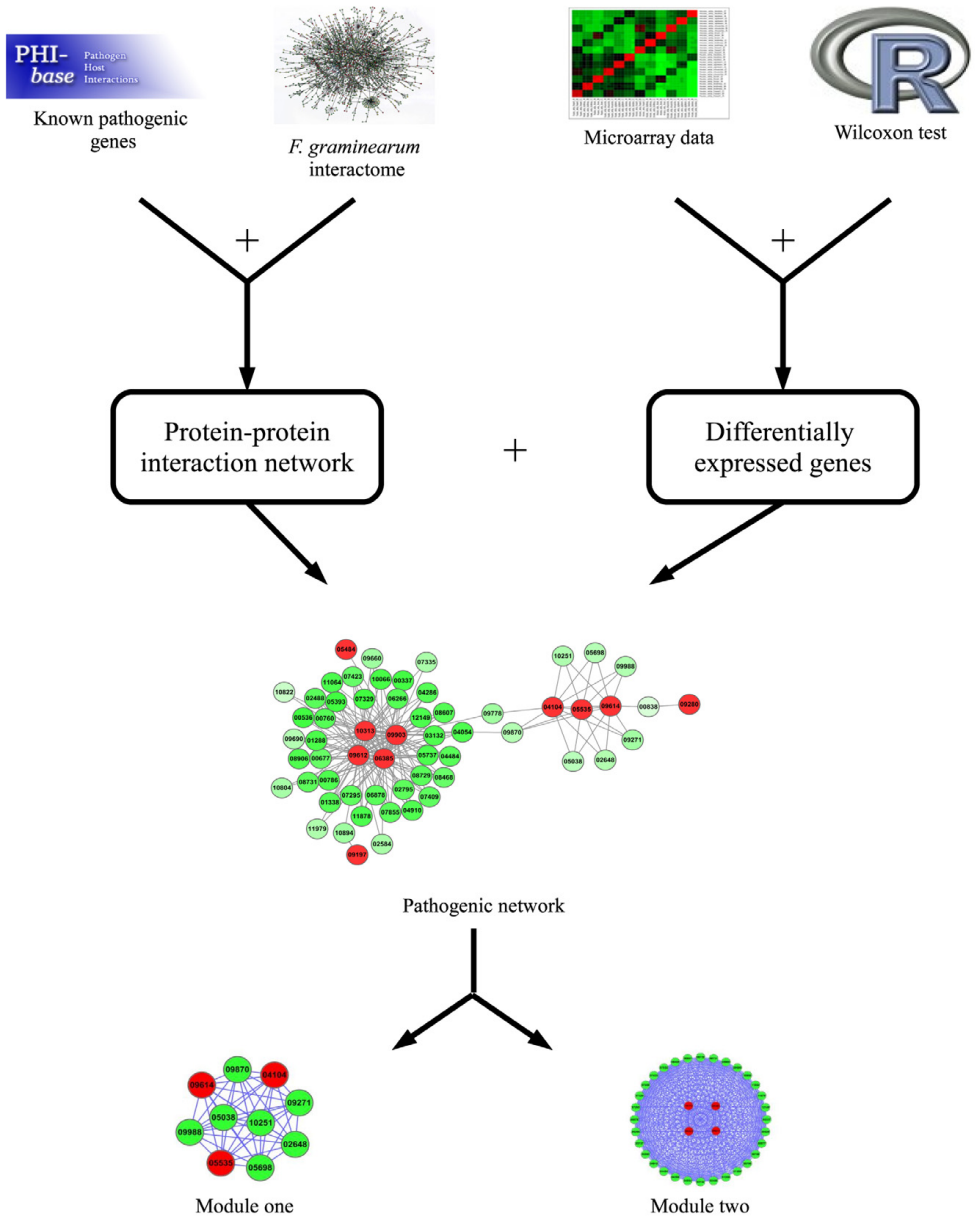
Flowchart of a novel network approach to predict pathogenic genes. The differentially expressed genes were identified first. Subsequently, a pathogenic network was extracted, and the network was mapped by differentially expressed genes that interact with at least two seed genes. In addition, the modules that consist of genes intensely interacting with each other were identified, and the genes in the modules are believed to be more likely to be pathogenic genes.

Figure 6 shows the flowchart of predicting pathogenic genes based on protein-protein interaction and gene expression data. There are some genes that have been identified as pathogenic genes deposited in PHI-base (http://www.phi-base.org/query.php), which contains manually curated genes proven to affect the outcome of pathogen-host interactions. There are 49 genes in total are found to be pathogenic genes of *F. graminearum* according to PHI-base and were downloaded for future studies. Since some *F. graminearum* genes from PHI-base have different names from those from Broad Institute, these genes were aligned against those obtained from Broad Institute using BLAST, and the best hit was found for each gene and the name was used as the one defined by Broad Institute. For example, PKS2 got its best hit of FGSG_04694. However, some genes in PHI-base do not have nucleic acid or animo acid sequences, which were then queried in MIPS FGDB database and the gene names were retrieved. For example, HMR1 was named as FGSG_09197.

The known pathogenic genes described above were then mapped to the protein-protein interaction network (PPIN) predicted by our previous work [8]. In this work, only the high-confidence protein interactions were used in PPIN, *i.e.* 27,102 interactions and 3,745 proteins. Consequently, there are 20 genes that can be mapped to PPIN of *F. graminearum* due to the incompleteness of PPIN, and these genes were treated as seed genes in sequel. Subsequently, a network was extracted from PPIN that consists of genes that interact with seed genes, where the genes were further required to be differentially expressed before and after the invasion of the pathogenic fungus. Therefore, the genes in the subnetwork are more possibly pathogenic genes. Furthermore, a smaller subnetwork that consists of genes interacting with at least two seed genes was extracted from previous network and regarded as pathogenic network, where the genes in pathogenic network are believed to be related to pathogenesis.

In addition, two intensely connected network modules were identified from the pathogenic network by employing maxClique, a tool in RBGL package [14] of Bioconductor, and each module is possibly a pathway. The functions of the genes in the network modules were investigated with the annotations from MIPS FGDB database. The pathogenic network and modules were visualized with Cytoscape (http://www.cytoscape.org/).

### Ranking of the pathogenic genes

To facilitate the biologists to choose more confident pathogenic genes from our predictions. Each gene was assigned a weight according to the interactions and co-expressions with seed genes, where a gene is more confident to be a pathogenic gene if it interacts and is co-expressed with more seed genes. The co-expression is evaluated by the *Pearson* correlation coefficients between our predicted pathogenic gene and seed genes based on all gene expression data, including those measured before and after *F. graminearum* invading its host.

With the correlation coefficients obtained above, the weight 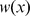 for each gene 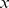 is defined as follows,
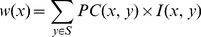
(1)Where 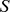 is the set of known pathogenic genes, 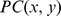 is the correlation coefficient between gene 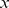 and gene 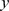, and 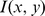 is an indication function, where 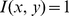 if protein 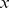 interacts with protein 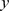 and 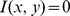 otherwise. The weight of each predicted pathogenic gene can illustrate the correlation between this gene and the seed genes. The higher the weight of one gene is, the more possible the gene is involved in pathogenic procedure.

### Properties of pathogenic network

To investigate the possible roles of the pathogenic genes predicted above, the network properties were investigated for all genes in the PPIN of *F. graminearum*, including degree, clustering coefficient and betweenness. These indices were calculated respectively for seed genes, predicted pathogenic genes and all genes in PPIN so that we can investigate the specific properties of the pathogenic genes.

The PPIN can be represented as an undirected network 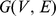, where 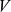 is the set of vertices and 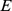 is the set of edges. The degree 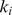 of a vertex 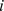 is the number of edges connected to that vertex, which can be computed as follows
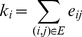
(2)where 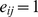 if there is an edge between node 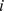 and node 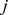 and 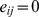 otherwise. The average degree of a network is the average of 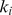 over all vertices in the network,
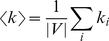
(3)The clustering coefficient of a vertex is an index that quantifies how close a vertex connects to its neighbors, and is defined as below
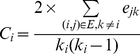
(4)Where 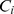 is the clustering coefficient for vertex 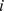, 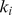 is the degree of vertex 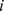, 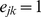 if 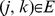 and 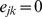 otherwise. The average clustering coefficient of a network is defined:
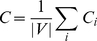
(5)


The Betweenness is one of the standard measures of node centrality, which is originally introduced to qualify the importance of a node in a social network. It is normally calculated as the fraction of the shortest paths between node pairs that pass through the node of interest [45].
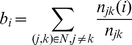
(6)where 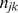 is the number of the shortest paths connecting nodes 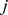 and 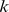, while 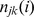 is the number of the shortest paths connecting 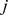 and 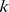 but passing through 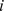.

### Identification of orthologous genes

To investigate whether our predicted pathogenic genes have already been confirmed in other pathogenic fungi, some pathogenic fungi genome were downloaded from Broad Institute, including *Magnaporthe grisea*, *Botrytis cinerea*, *Ustilago maydis*, *Venturia inaequalis*, *Rhynchosporium secalis*, and *Cryphonectria parasitica*. The orhtologs of *F. graminearum* proteins were identified in other pathogenic fungi by utilizing Inparanoid [27]. Especially, the orthologs in other pathogenic fungi were investigated to see whether they have been already verified to be pathogenic genes using the annotations from PHI-base. If the orthologs of our predicted pathogenic gene are found to be annotated as pathogenic genes in PHI-base, the predicted pathogenic gene is believed to be potential pathogenic gene of *F. graminearum*.

### Statistical analysis of prediction results

To see the significance of the predicted modules, a significance score (SS) is defined for each module as the geometric mean of 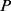-values accompanying the nodes in one module, where the 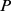-value for each node is obtained by the Mann-Whitney Wilcoxon test based on gene expression data before and after the invasion of the pathogen. Since all the genes are differentially expressed in our background network, it does not necessarily mean that the genes in one module are more differentially expressed, *i.e.* with smaller 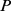-values. On the other hand, a set of genes are more possibly involved in pathogenesis if these genes are closely interacted and more differentially expressed because pathogenesis generally involves a set of concert-acting genes. Therefore, the SS defined here can evaluate the significance of one module.

**Figure 7:**
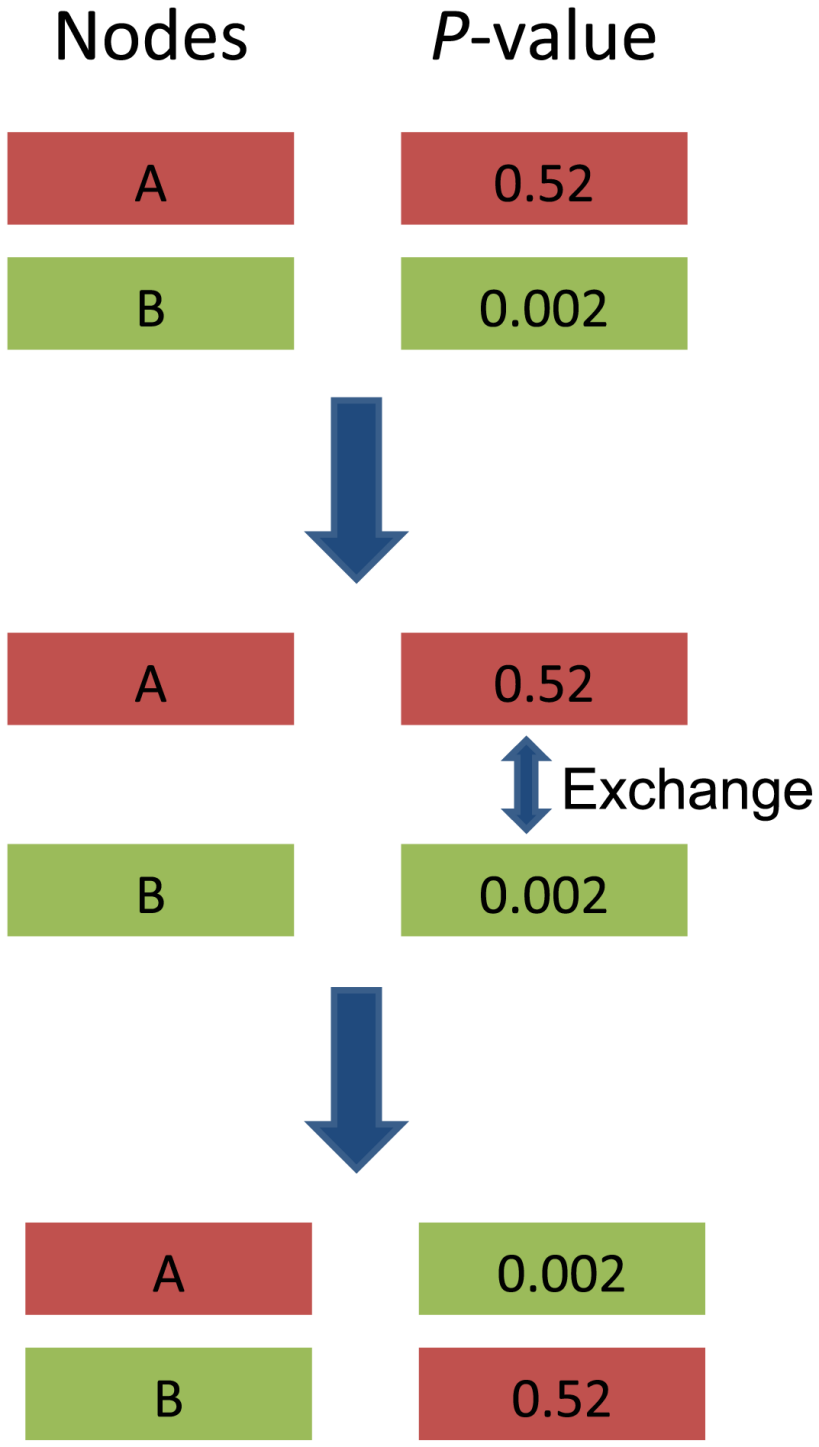
Randomization of network labels. A and B denote two differentially expressed genes in the network, where each gene is labeled with a differential expression derived *P*-value, *i.e.* 0.005 for gene A and 0.002 for gene B. After randomization, the labels of the two genes are exchanged, *i.e.* 0.002 for gene A and 0.005 for gene B.

To see the statistical significance of the two predicted modules, a 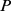-value was respectively obtained for each module by using an empirical randomization test procedure that preserves the interactions among genes. Firstly, the 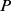-values of the genes in the network are randomly shuffled and each gene will get a new 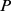-value after shuffling (Shown in Figure 7). Secondly, the SSs for the two modules are recalculated after the 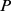-value labels are shuffled and these are regarded as null distribution of SSs. Thirdly, the randomization is repeated for 10000 times. Fourthly, the 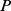-value for a module is defined as the probability that one module can be detected in randomization procedure with smaller SS than that of our predicted module.

## Supporting Information

Figure S1 The module predicted by MCODE. The red nodes are seed nodes, and the green nodes are non-seed nodes, this module includes module two we predicted.
(4.16 MB TIF)

Table S1 Network modules consist of differentially expressed genes that intensely interact with each other. The functions of the genes in the modules were downloaded from MIPS FGDB, and the known pathogenic genes were marked in bold.
(0.05 MB PDF)

Table S2 GeneName is all of the genes which is connected with seed genes, GeneWeight is the weight for every nodes, DiseaseNum is the number of seed genes which are connected by genes in GeneName column.
(0.03 MB XLS)
