# Supplementary figures and images for: A Network Approach to Predict Pathogenic Genes for Fusarium graminearum
Source: PLoS One. 2010 Oct 4;5(10):e13021. doi: 10.1371/journal.pone.0013021 (PMC2949387; doi:10.1371/journal.pone.0013021)

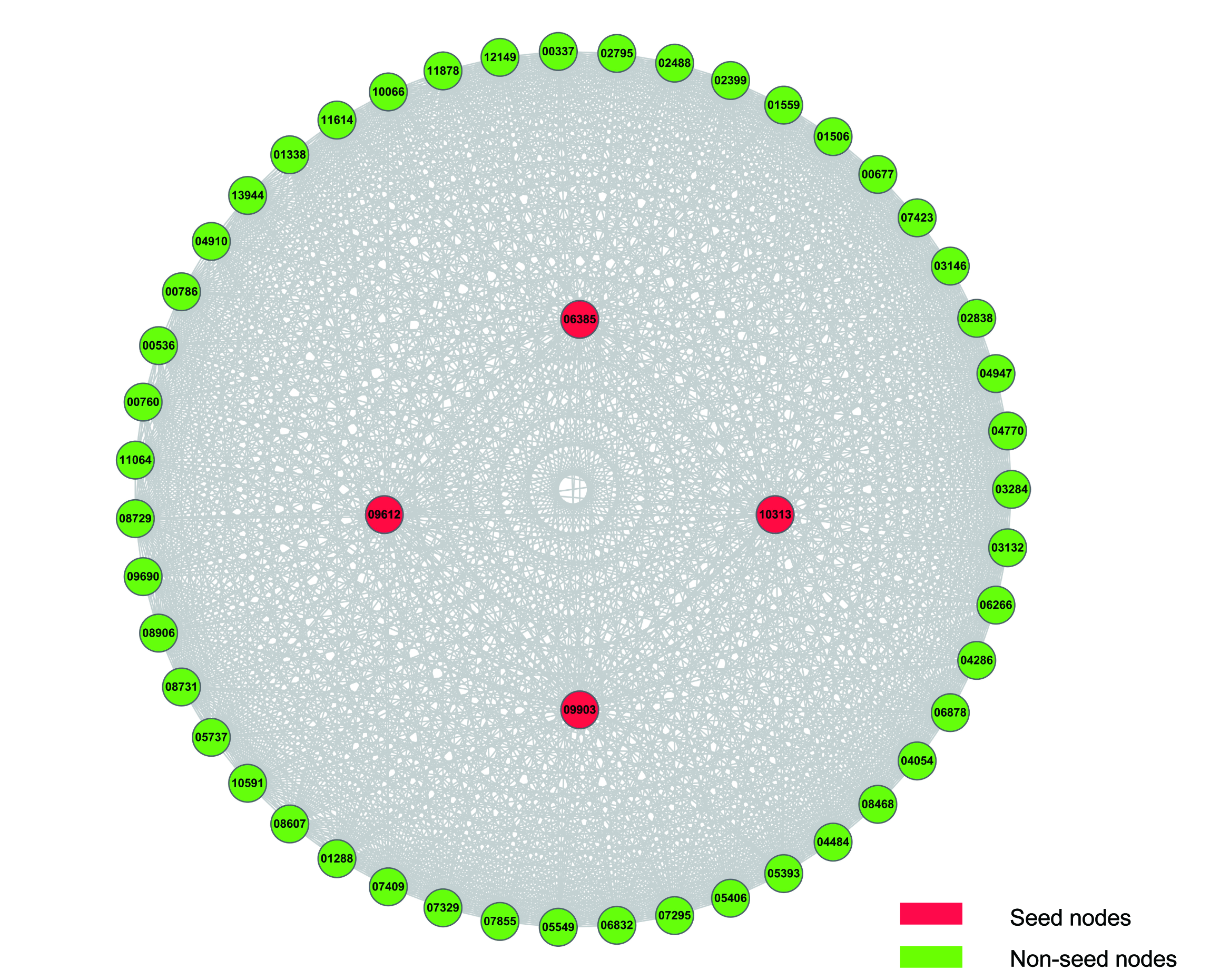

Supplement: Figure S1 — The module predicted by MCODE. The red nodes are seed nodes, and the green nodes are non-seed nodes, this module includes module two we predicted. (4.16 MB TIF) [file pone.0013021.s001.tif]
