# Supplementary material for: A Network Approach to Predict Pathogenic Genes for Fusarium graminearum
Source: PLoS One. 2010 Oct 4;5(10):e13021. doi: 10.1371/journal.pone.0013021 (PMC2949387; doi:10.1371/journal.pone.0013021)
Supplement: Table S1 — Network modules consist of differentially expressed genes that intensely interact with each other. The functions of the genes in the modules were downloaded from MIPS FGDB, and the known pathogenic genes were marked in bold. (0.05 MB PDF) [file pone.0013021.s002.pdf]

Table S1 - Pathogenic network modules

Network modules consist of differentially expressed genes that intensely interact with each other. The functions of the genes in the modules were downloaded from MIPS FGDB, and the known pathogenic genes were marked in bold.

Network modules	Genes	MIPS annoations
Module one	**FGSG_04104**	probable guanine nucleotide-binding protein beta subunit
	**FGSG_05535**	probable G protein alpha chain
	**FGSG_09614**	probable G protein alpha subunit GNA-3
	FGSG_09271	probable SEC13 - protein transport protein
	FGSG_05698	probable WD40-repeat protein (notchless protein)
	FGSG_09870	probable CPC2 protein
	FGSG_09988	probable G protein alpha chain
	FGSG_10251	probable LST8 protein
	FGSG_02648	probable U5 snRNP-specific 40 kD protein (novel WD-40 repeat protein)
	FGSG_05038	probable nuclear migration protein
Module two	**FGSG_06385**	pathogenicity MAP kinase 1
	**FGSG_10313**	MAP kinase
	**FGSG_09903**	probable MAP kinase kinase
	**FGSG_09612**	probable osmotic sensitive-2 protein (putative mitogen-activated protein (MAP) kinase homolog)
	FGSG_10066	probable casein kinase I CKI2
	FGSG_00337	probable calmodulin-dependent protein kinase
	FGSG_04484	related to cyclin-dependent kinase chain SRB10
	FGSG_00760	related to single-stranded DNA binding protein
	FGSG_08906	related to serine/threonine-specific protein kinase
	FGSG_11878	probable cutinase negative acting protein
	FGSG_11064	related to glycine-rich RNA-binding protein
	FGSG_08468	probable CDC28 - cyclin-dependent protein kinase
	FGSG_08607	probable casein kinase II beta subunit CKB1
	FGSG_00677	probable protein kinase CK2 catalytic subunit CK2 alpha-3
	FGSG_01338	related to 26s proteasome subunit p28
	FGSG_00786	related to serine/threonine-protein kinase
	FGSG_07295	probable MAP kinase kinase
	FGSG_02488	related to Dis1-suppressing protein kinase DSK1
	FGSG_12149	probable serine/threonine-specific kinase KSP1
	FGSG_01288	probable casein kinase II beta subunit (regulator of circadian clock protein FRQ)
	FGSG_04286	related to small nuclear ribonucleoprotein snRNP U1A
	FGSG_08731	probable casein kinase-1 HHP1
	FGSG_07423	probable cyclin-dependent ser/thr protein kinase KIN28
	FGSG_07409	related to cyclin dependent kinase C
	FGSG_08729	related to cAMP-dependent protein kinase catalytic subunit
	FGSG_06266	related to cyclophilin
	FGSG_09690	probable peptidylprolyl isomerase (FK506-binding protein homolog)
	FGSG_06878	probable CMK1 - Ca2+/calmodulin-dependent ser/thr protein kinase type I
	FGSG_07855	probable CDC7 - protein kinase
	FGSG_05393	probable PHO85 - cyclin-dependent protein kinase
	FGSG_07329	probable glycogen synthase kinase 3 alpha
	FGSG_04054	related to VHS1 - protein kinase involved in G1/S transition
	FGSG_04910	probable cyclin-dependent kinases regulatory subunit CKS1
	FGSG_00536	conserved hypothetical protein
	FGSG_03132	probable CDC28 - cyclin-dependent protein kinase
	FGSG_05737	related to polyadenylate-binding protein
